# Development of a Highly Sensitive Glycan Microarray for Quantifying AFP-L3 for Early Prediction of Hepatitis B Virus–Related Hepatocellular Carcinoma

**DOI:** 10.1371/journal.pone.0099959

**Published:** 2014-06-13

**Authors:** Chen-Shiou Wu, Teng-Yu Lee, Ruey-Hwang Chou, Chia-Jui Yen, Wei-Chien Huang, Chung-Yi Wu, Yung-Luen Yu

**Affiliations:** 1 The Ph.D. Program for Cancer Biology and Drug Discovery, China Medical University, Taichung, Taiwan; 2 Graduate Institute of Clinical Medical Science, China Medical University, Taichung, Taiwan; 3 Graduate Institute of Cancer Biology, and Center for Molecular Medicine, China Medical University, Taichung, Taiwan; 4 Genomics Research Center, Academia Sinica, Taipei, Taiwan; 5 Department of Medicine, Chung Shan Medical University, Taichung, Taiwan; 6 Division of Gastroenterology, Taichung Veterans General Hospital, Taichung, Taiwan; 7 Department of Biotechnology, Asia University, Taichung, Taiwan; 8 Graduate Institute of Clinical Medicine, National Cheng Kung University, Tainan, Taiwan; 9 Division of Hematology/Oncology, Department of Internal Medicine, National Cheng Kung University Hospital, Tainan, Taiwan; CRCL-INSERM, France

## Abstract

The α-fetoprotein fraction L3 (AFP-L3), which is synthesized by malignant cells and incorporates a fucosylated oligosaccharide, has been investigated as a diagnostic and prognostic marker for hepatocellular carcinoma (HCC). Quantification of AFP-L3 by conventional enzyme-linked immunosorbent assay (ELISA) has not always produced reliable results for serum samples with low AFP, and thus we evaluated the clinical utility of quantifying AFP-L3 using a new and highly sensitive glycan microarray assay. Sera from 9 patients with chronic hepatitis B and 32 patients with hepatitis B virus (HBV)-related HCC were tested for AFP-L3 level using the glycan microarray. Additionally, we compared receiver operator characteristic curves for the ELISA and glycan microarray methods for determination of the AFP-L3: AFP-L1 ratio in patient samples. This ratio was calculated for 8 HCC patients who underwent transarterial embolization therapy pre- or post-treatment with AFP-L3. Glycan microarrays showed that the AFP-L3 ratio of HBV-related HCC patients was significantly higher than that measured for chronic hepatitis B patients. Overall parameters for estimating AFP-L3% in HCC samples were as follows: sensitivity, 53.13%; specificity, 88.89%; and area under the curve, 0.75. The elevated AFP-L3% in the 8 patients with HBV-related HCC was strongly associated with HCC progression. Following one month of transarterial embolization therapy, the relative mean AFP-L3% decreased significantly. In addition, we compared Fut8 gene expression between paired tumor and non-tumor tissues from 24 patients with HBV-related HCC. The Fut8 mRNA expression was significantly increased in tumorous tissues in these patients than that in non-tumor tissue controls. Higher expression of Fut8 mRNA in tumorous tissues in these patients was associated with poor differentiation than well and moderate differentiation. Our results describe a new glycan microarray for the sensitive and rapid quantification of fucosylated AFP; this method is potentially applicable to screening changes in AFP-L3 level for assessment of HCC progression.

## Introduction

Hepatocellular carcinoma (HCC) is the sixth most common malignancy and the third most common cause of cancer-related death worldwide [1], and a large proportion of cases are diagnosed as advanced-stage HCC [2]. Serological tests for α-fetoprotein (AFP) and use of ultrasonography have been suggested as HCC surveillance methods, and when ultrasound is not readily available, current guidelines recommend checking AFP levels every 6 months in high-risk populations [3]. However, the sensitivity and specificity of AFP are suboptimal for HCC diagnosis, and AFP can be elevated in patients with both HCC and chronic liver disease [4]–[6]. Although recent advances in dynamic imaging techniques, including computed tomography and magnetic resonance imaging, have facilitated the detection of small and early-stage HCC [7]–[9], the relative high cost and risks associated with these techniques limit their use. Because patients with advanced HCC generally have a poor prognosis, detection of early-stage HCC is the best strategy to improve outcomes [10], [11]. In this regard, there is urgent need to identify more sensitive and reliable serum biomarkers for detection of HCC.

Recent studies have described a core fucosylated AFP (AFP-L3) that is the product of α1-6-fucosyltransferase (Fut8) in the presence of GDP fucose [12]–[14]. AFP-L3 level is reported as the percentage of AFP-L3 divided by the total AFP in the sample [15]. Previous studies have identified a 10% AFP-L3 level as the cut-off for diagnosis of HCC [15], and this criterion enables earlier detection of incident or recurrent HCC compared with use of imaging techniques [16]. Therefore, AFP-L3 is considered more specific than AFP for diagnosis of HCC [17]–[19], and monitoring AFP-L3 level increases the detection of small HCCs [5].

The basic technique for lectin-antibody enzyme immunoassay of AFP-L3 has already been established [20]. Owing to limitations in instrument sensitivity, however, the conventional method for measurement of AFP-L3, which involves use of reference lectin-affinity electrophoresis coupled with antibody-affinity blotting or a liquid-phase binding assay, is not always reliable [21]. During the last decade, the development of glycan microarray assays has enabled the highly sensitive and high-throughput analysis of glycoproteins, contributing to significant advances in glycomics [22]. Glycan microarrays are not only a powerful tool for basic research but also a promising technique for medical diagnosis and detection of pathogens and cancers. Therefore, we estimated AFP-L3 levels using a newly developed method involving glycan microarray technology, and then we evaluated associations between the resultant assay values and clinical features such as HCC development to determine the usefulness of this measure for HCC detection.

## Results

### Construction of a microarray of glycan fractions of α-fetoprotein (AFP-L1 and AFP-L3)

**Figure 1:**
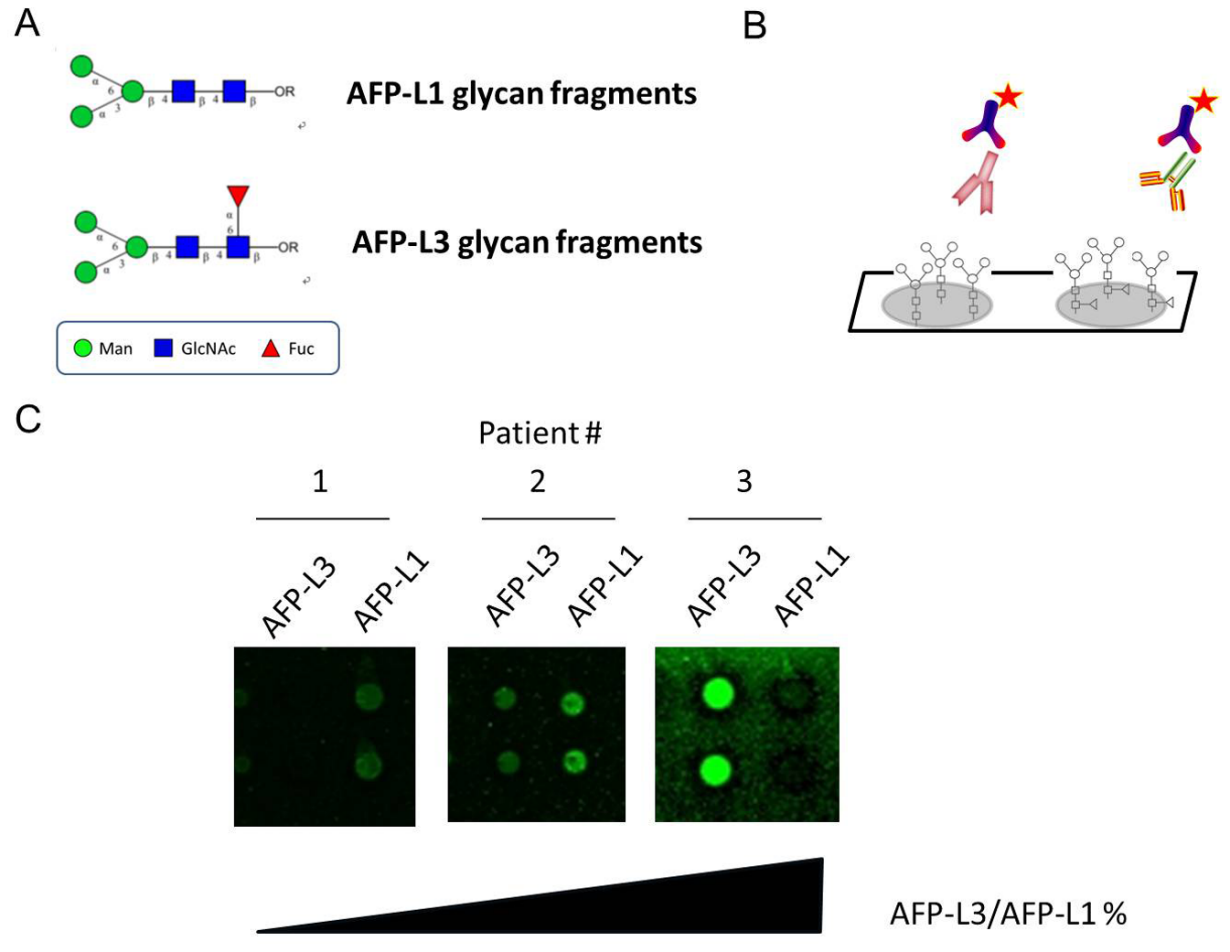
The glycan microarray utilized in the study. (A) Schematic of chemically synthesized oligosaccharides (AFP-L1 and AFP-L3 fragments). (B) Illustration of the characterization and quantification of the oligosaccharides immobilized on the supporting surface. Cy3-labeled antibody is added and binds to the captured analyte. (C) Readout by fluorescence scanner.

**Figure 1A** shows chemical structures of chemically synthesized AFP-L1 and AFP-L3. Samples for analysis were then added, and any analyte present was bound by the immobilized glycan. Next, Cy3-labeled antibody (detection antibody) was added and bound to the captured analyte (**Figure 1B**). Fluorescence from each slide was measured at 532 nm (for Cy3-conjugated secondary antibody) with a microarray chip reader (arrayWoRx microarray reader). Representative slide images obtained from a fluorescence scan are shown in **Figure 1C**.

### Detection of total AFP and total AFP-L3 by enzyme-linked immunosorbent assay (ELISA)

**Figure 2:**
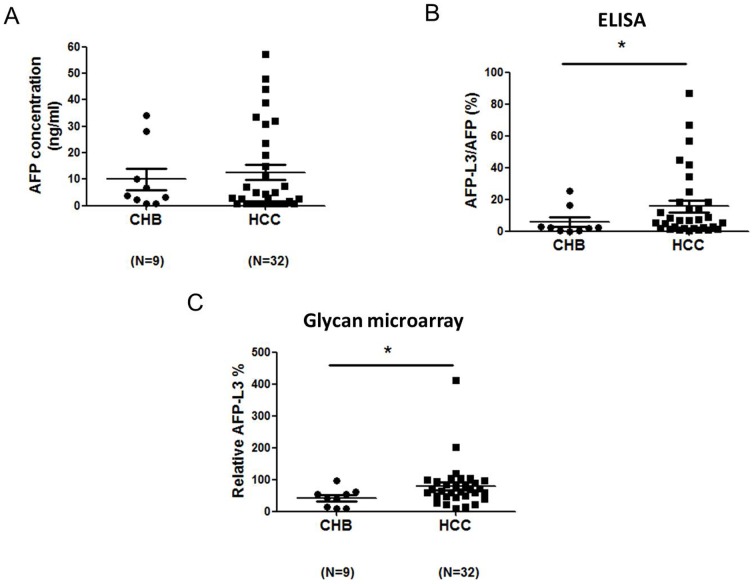
Serum levels of AFP-L3 in patients with CHB and HCC. (A) AFP concentrations measured by ELISA. (B) ELISA for AFP-L3% in sera from patients with CHB and HCC (*P<0.05). (C) Glycan microarray for AFP-L3% in the sera from patients with CHB and HCC (*P<0.05).

The AFP concentration in the serum of a healthy adult is typically <20 ng/mL [23]. Among the patients with chronic hepatitis B (CHB), the AFP value exceeded 20 ng/mL for 2 patients and was <20 ng/mL for 7 patients. Among the patients with HCC, the AFP value exceeded 20 ng/mL for 8 patients and was <20 ng/mL for 24 patients (**Figure 2A**). These results showed that a large proportion of HCC patients had a normal AFP value, underscoring the difficulty of diagnosing the disease using ELISA. The low specificity of AFP has been a cause of concern for its use as an HCC marker.

### Determining AFP-L3 percentage (AFP-L3%) by ELISA and glycan microarray, respectively

We next investigated the reliability of data obtained for AFP-L3 levels and examined whether AFP-L3 could be quantified by the glycan microarray. Although similar results were obtained when using ELISA for assessment of AFP-L3% in the CHB and HCC groups, a significantly higher AFP-L3% was measured for HCC patients when using the microarray assay (5.72±2.98.2% vs. 15.76±4.65%, *P* = 0.045) (**Figure 2B**). The glycan microarray also revealed a significantly higher AFP-L3% for HCC patients than for CHB patients (2.59±0.42% vs. 1.22±0.33%, *P* = 0.014; **Figure 2C**).

### Establishment of optimal cut-off values for AFP-L3% by ELISA and glycan microarray, respectively

**Figure 3:**
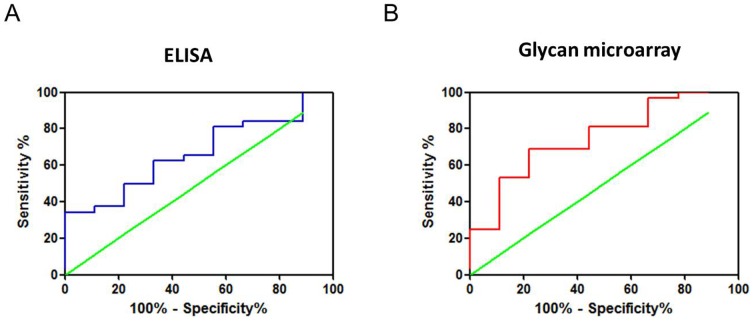
Receiver–operator characteristic curves for AFP-L3% in patients. (A) ELISA revealed an AFP-L3% cut-off value of 1.873% as a tumor marker for HCC, which had a sensitivity of 50% and specificity of 77.78%. Under these conditions, the area under the receiver–operator characteristic curve was 0.6667 (0.4856–0.8477, 95% CI, *P* = 0.13). (B) The glycan microarray revealed an AFP-L3% cut-off value of 0.6388 as a tumor marker for HCC, which had a sensitivity and specificity of 53.13% and 88.89%. Under these conditions, the area under the receiver–operator characteristic curve was 0.75 (0.5727–0.9273, 95% CI, *P*<0.05).

A receiver–operator characteristic curve was generated and used to determine the optimal cut-off value of AFP-L3% using data obtained from the 41 samples in which the AFP-L3 level was above the detection limit. When using ELISA and a cut-off value of 2.67%, the mean area under curve value was 0.67 (0.49–0.85, 95% confidence interval (CI), *P* = 0.13; **Figure 3A**). The sensitivity and specificity of the glycan microarray assay were greatest when using a cut-off value of 0.6388 and a mean area under curve value of 0.75 (0.57–0.93, 95% CI, *P* = 0.02; **Figure 3B**).

### Determinationof AFP-L3% and AFP values at different times during treatment of HCC patients

**Figure 4:**
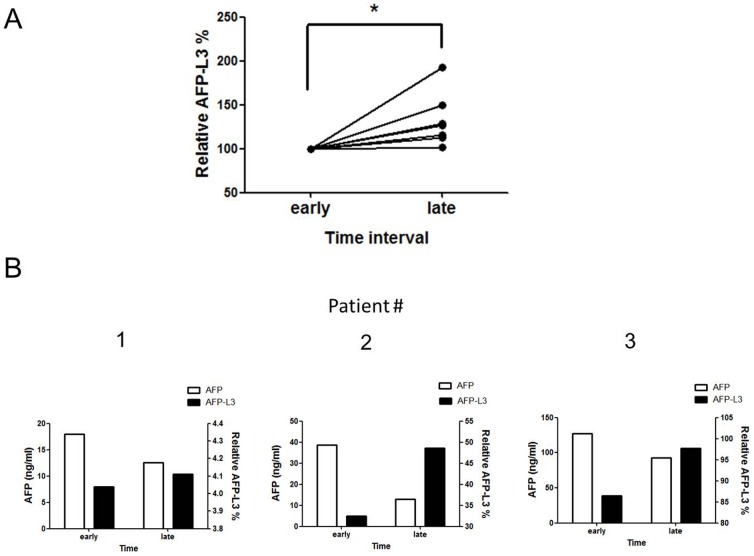
Values for reactivity of AFP-L3% and AFP at different times for patients with HCC. (A) Dynamic changes of AFP-L3% in each HCC patient. The results of the assay showed that AFP-L3% level had increased significantly at point in late time (n = 8; *P<0.05). (B) AFP-L3% and AFP values at different time points for patients with HCC.

Ultrasound imaging and AFP values are typically used to diagnose and monitor HCC. By the time abnormalities are identified by these methods, however, HCC has typically reached an advanced stage. We therefore examined whether the AFP-L3 values for an individual patient differed at different points in time and whether these values could be used as an auxiliary diagnostic marker. Patients in the HCC group had been treated with drugs for prolonged periods prior to the first assessment of AFP-L3%. The second study of AFP-L3% was conducted >6 months later, during which the patients had continued to receive drug therapy. The results of the second assay showed that AFP-L3% assay-based reactivity had increased significantly (*P* = 0.03) (**Figure 4A**). This result indicated that patient AFP values had declined to normal levels following prolonged drug therapy; however, their AFP-L3% tended to increase over time (**Figure 4B**).

### Relationship of pre- and post-treatment AFP-L3% values for patients undergoing transarterial embolization (TAE) therapy

**Figure 5:**
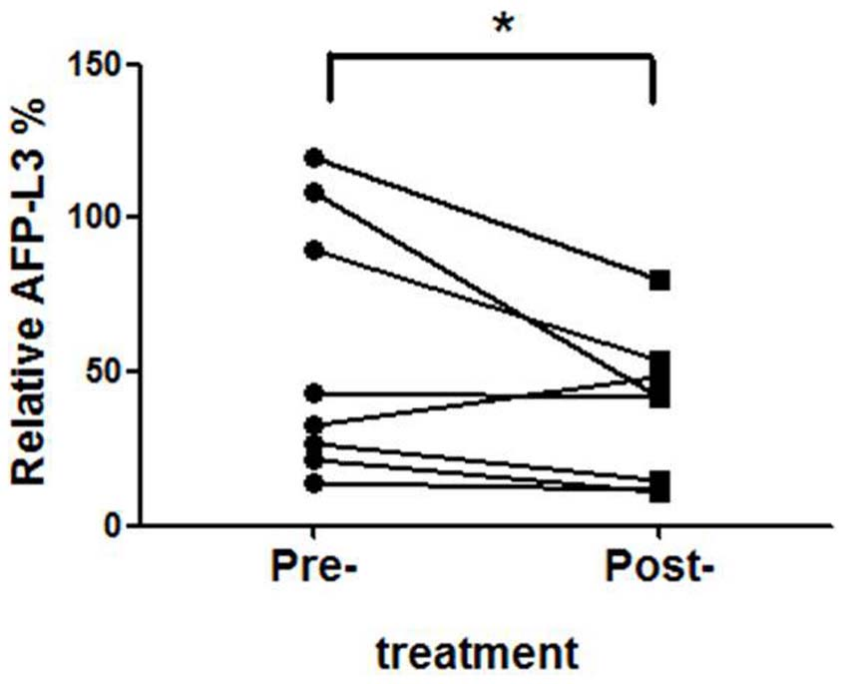
Comparison of AFP-L3% at pre- and post-TAE treatment. Post-treatment AFP-L3% values (0.38±0.09) were significantly lower than the pre-treatment point time (0.57±0.16) in different patients (n = 8; *P<0.05).

TAE is the treatment of choice for advanced HCC to control or even induce tumor shrinkage. We next compared the pre- and post-treatment values for AFP-L3% in the eight patients treated with TAE. Surprisingly, post-treatment point time AFP-L3% values (0.38±0.09) were significantly lower than the pre-treatment point time (0.57±0.16) (*P* = 0.04; **Figure 5**).

### The Fut8 mRNA levels for non-tumor (adjacent) liver tissues and tumor tissue were evaluated by qPCR

**Figure 6:**
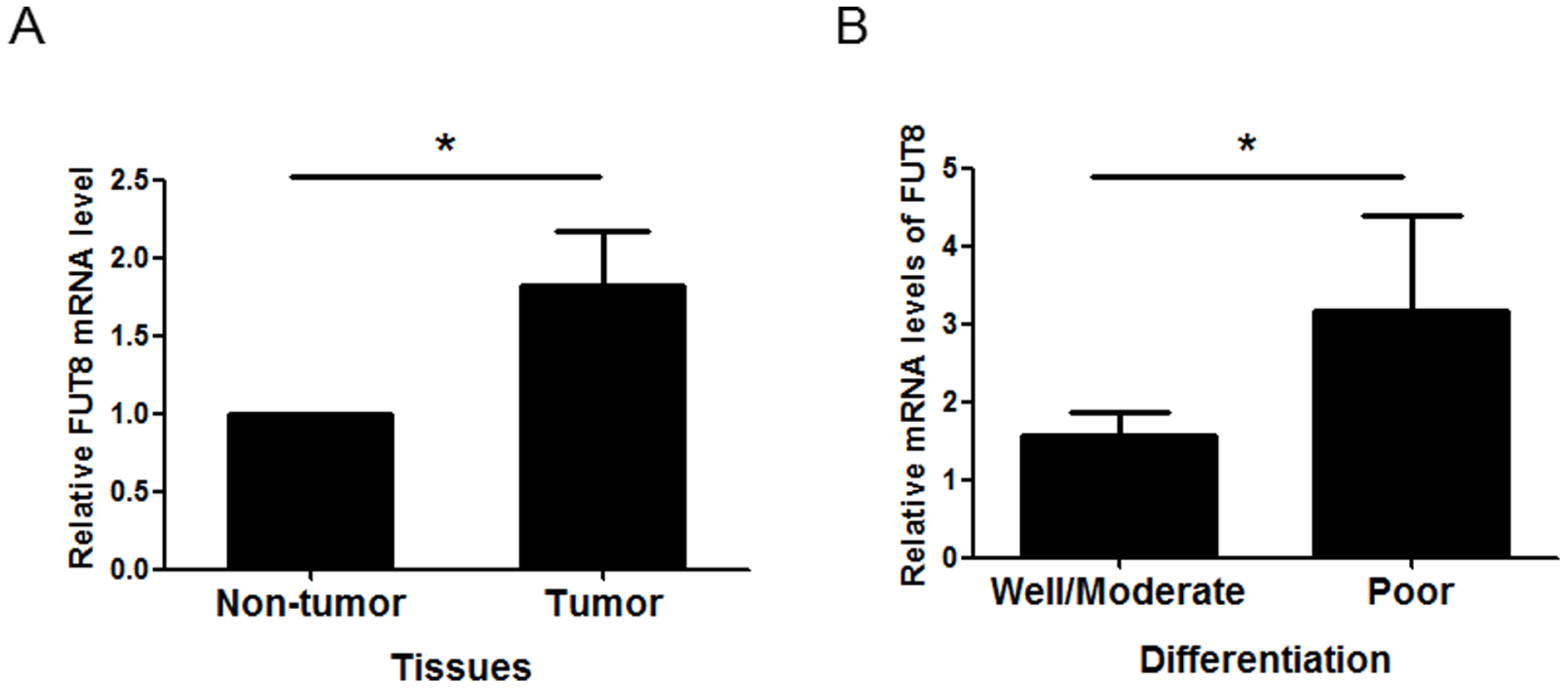
Fut8 expression is increased in HCC tumor tissues. (A) The Fut8 mRNA levels were evaluated in HCC tumor tissue compared normal liver tissue from the same patients (n = 24; *, *P*<0.05). (B) Association between Fut8 levels and histologic grade (*, *P*<0.05).

Fut8 is involved in the biosynthesis of AFP-L1 to AFP-L3. To determine the levels of Fut8 in tumor, we analyzed the expression of Fut8 in 24 pairs of HCC tumor tissues and their corresponding adjacent non-tumor liver tissue. A significant increase in Fut8 expression, with a fold change about 1.82, was observed in tumor tissues with respect to non-tumor liver tissues (*P* = 0.01; **Figure 6A**).

### Fut8 expression is associated with histological grade of HCC

We next asked if Fut8 expression is affected during liver cancer progression. In a subset of 24 paired samples (tumor tissue compared with non-tumor tissue from the same patient), we found a statistically significant enhance in Fut8 expression in poor differentiation as compared with well and moderate (*P* = 0.04; **Figure 6B**). Therefore, these results provide the relation between Fut8 expression and HCC progression and point to a promising direction for the prognosis and therapy of HCC.

## Discussion

In this study, we first investigated the clinical utility of AFP-L3 via a newly developed and highly sensitive method (the glycan microarray) using serum samples from patients with CHB and HCC. The glycan microarray can be used to detect anti-AFP-L3 that recognizes glycans associated with AFP. Such glycan microarrays are being developed to decode the informational content of the glycome [24] and are important tools for qualitative and quantitative studies of glycoproteins and glycoenzyme specificities using high-throughput systems.

It is well known that AFP-L3 concentration correlates well with AFP; however, AFP-L3% is not correlated with AFP [25], [26]. AFP-L3% is a marker that is independent of AFP. We found that the levels of AFP-L3–specific IgG were significantly higher in HCC patients than in CHB patients. Additionally, consistent with previous studies [25], elevation of the AFP-L3 ratio was independent of increases in total AFP in HCC patients. Although prolonged observations will be required to clarify whether the AFP-L3 ratio is useful for predicting HCC, our findings suggest that this ratio is useful for early detection of HCC compared with CHB, even in subjects with serum AFP serum levels <20 ng/mL. Taketa et al. [25] have reported that AFP-L3 values elevated above the cutoff value of 15% with an average of 4.0±4.9 months before the detection of HCC by imaging techniques. Sato et al. [27] also have demonstrated that lectin-reactive AFP elevated 3–18 months before the detection. Also, an increase in the AFP-L3 ratio prior to detection of HCC by various advanced imaging modalities may contribute to the more precise identification of chronic liver disease in patients with a relatively high risk of HCC [28]. In this study, we demonstrated that the glycan microarray assay has great clinical utility for detecting HCC changes in patients with low total AFP concentrations after treatment for HCC.

We found that the preoperative AFP-L3 ratio was strongly correlated with the presence of HCC, and thus this ratio may be useful for predicting HCC prognosis. The AFP-L3 ratio provides information complementary to total AFP level that can be used for the early recognition of malignant liver tumors and during follow-up of patients after therapy [17]. Our data are the first to show changes of AFP-L3% both before and after TAE therapy, and we also observed that the AFP-L3% was significantly reduced after palliative treatment. HCC patients with an increased proportion of AFP-L3 have a poorer prognosis and thus should receive more aggressive treatment and follow-up. Additionally, many studies have investigated the role of AFP-L3 as a marker during surveillance of patients at risk for HCC [15], [29]–[32].

Core-fucosylation by Fut8 resulted in the production of AFP-L3. In the current study, we report that the expression of Fut8 is significantly up-regulated in tumor tissues of patients with HCC, and the up-regulation of Fut8 is associated with poorly differentiated cancer in patients with HCC. According to these findings, we speculate that Fut8 is a key factor in promoting malignant progression of tumors. HCC initially develops as well-differentiated HCC, and then progresses to moderately- to poorly-differentiated HCC via a process of dedifferentiation. The clinical relevance of Fut8 overexpression in HCC has been elucidated [33]. Recently, knocking down Fut8 is reported to attenuate TGF-β–induced EMT in human renal proximal tubular epithelial cells [34], suggesting an essential role for core fucosylation in EMT. Our results suggest that up-regulation of Fut8 is HCC-related; we thus suggest that Fut8 is a potential biomarker for predicting malignant progression of HCC. Other studies have shown AFP-L3 fraction levels have been associated with portal vein invasion and advanced tumoral stage, a fact that prevents the usage of these markers for early detection [35], [36]. Our results indicate that the presence of anti-AFP-L3 antibodies in blood circulation is associated with an increased activity of Fut8 activity in HCC. Both tissue Fut8 levels and serum anti-AFP-L3 antibodies are potential prognostic markers for patients with operable HCC.

In conclusion, our study demonstrates that measuring AFP-L3 level is superior to AFP level for differentiating between HCC and CHB. Additionally, AFP-L3 detection by glycan microarray shows great potential clinical value for the early diagnosis of HCC.

## Materials and Methods

### Ethics statement

The study protocol was approved by the Institutional Review Board of Taichung Veterans General Hospital in Taichung, Taiwan (assign number: CE11041). Additionally, written informed consent was obtained from participants for the use of their blood in this study. Tissue samples from patients with HBV-related HCC were collected at the National Cheng Kung University Hospital in Tainan, Taiwan. Before commencing the study, approval was obtained from the Institutional Review Board of National Cheng Kung University Hospital (assign number: ER-99-176) and informed written consent was obtain from each individual.

### Serum samples

Serum samples were collected from patients with CHB (n = 9) and hepatitis B virus–related HCC (n = 32) at the Taichung Veterans General Hospital in Taichung, Taiwan. Samples were encrypted to protect patient confidentiality and were used in accordance with a protocol approved by the Institutional Review Board of Human Subjects Research Ethics Committee. The patients were diagnosed using a combination of data (clinical, laboratory, and imaging findings and/or biopsy). All patient samples were stored at −20°C until use.

### General methods

NEXTERION slide H was purchased from SCHOTT North America. The coating on the SCHOTT NEXTERION Slide H consisted of a crosslinked, multicomponent polymer layer activated with N-hydroxysuccinimide esters to provide covalent immobilization of amine groups. The general procedure for the synthesis of glycans was conducted as reported [24], [37], [38].

### Glycan microarray fabrication

The glycan array that we used was non-commercial products and was mainly synthesized by Dr. Chung-Yi Wu laboratory [27], [33]. Microarrays were printed (BioDot; Cartesian Technologies) with a robotic pin (SMP3; TeleChem International) with a deposition of **≈**0.7 nL of various concentrations of amine-containing glycans in printing buffer (300 mM phosphate buffer [pH 8.5] containing 0.005% Tween-20) from a 96-well microtiter plate onto NHS-coated glass slides. The slides were spotted with 50 uM solutions of each AFP-L1 and AFP-L3 fragments, with two rows from bottom to top and two vertical replicates in each subarray. Printed slides were allowed to react in an atmosphere of 80% humidity for 1 h followed by desiccation overnight. The slides were stored at room temperature in a desiccator until use. Before the binding assay, the slides were blocked with ethanolamine (50 mM ethanolamine in 50 mM borate buffer [pH 9.2]) and then washed twice with water and phosphate buffer saline (PBS) (pH 7.4). The general procedure for the manufacture of arrays was conducted as reported [39].

### Microarray analysis of serum samples

Serum samples were diluted 1∶20 with a buffer consisting of PBS (pH 7.4) containing 0.05% Tween 20 and 3% BSA and applied to the grid of each glycan microarray. The microarrays were then incubated in a humidified chamber with shaking for 1 h. The microarray slides were then washed three times each with PBS (pH 7.4) containing 0.05% Tween 20, PBS, and water. Next, Cy3-conjugated goat anti–human IgG (Jackson ImmunoResearch, Jackson, ME, USA) was added to the slides as described above, and the slides were incubated under a coverslip in a humidified chamber with shaking for 1 h. The slides were washed three times each with PBS (pH 7.4) containing 0.05% Tween 20, PBS, and water, and then dried. The slides were scanned at 532 nm (for Cy3-conjugated secondary antibody) with a microarray fluorescence chip reader (arrayWoRx microarray reader, Applied Precision, Issaquah, WA, USA) [27], [33].

### Measurement of AFP and AFP-L3 by ELISA

Serum AFP and AFP-L3 concentrations were determined using a commercially available ELISA kit (USCN Life Science Inc., Wuhan, China).

### Quantitative reverse transcriptase–PCR (qPCR)

Total RNA was extracted with TRIzol reagent (Invitrogen, Grand Island, NY) according to the manufacturer's instructions. To examine the expression, qPCR was performed by the LightCycler 480 apparatus (Roche) according to the manufacturer's instructions using FastStart DNA Master SYBR Green (Roche). β-actin was used as an endogenous control. Double-stranded DNA specific expression was tested by the comparative Ct method using 2^−ΔΔCt^ [39]. Primers were designed using Primer3 software (http://frodo.wi.mit.edu/). The primer sets were as follows: Fut8: 5′-ACCAAGAAGCTTGGCTTCAA-3′ (forward), and 5′-TTTGTCCACTTGCATTCTGC-3′ (reverse); β-actin: 5′- GGACTTCGAGCAAGAGATGG-3′ (forward), and 5′-AGCACTGTGTTGGCGTACAG-3′.

### Data analysis

GenePix Pro software (Axon Instruments) was used to analyze fluorescence data. The local background was subtracted from the signal for each antibody spot, and the “medians of ratios” from replicate spots were averaged within the same array. The ratio of anti-AFP-L3:anti-AFP-L1 in sera was calculated by dividing the mean relative binding intensity for glycan replicates by the mean relative binding intensity for AFP-L1 replicates, and the results are expressed as percentages. Finally, statistical analysis was performed using an unpaired Student's *t*-test. Data were analyzed using Prism 5 software (GraphPad Software, Inc.).
